# CAECENET: An automatic system processing photometer and ceilometer data from different networks to provide columnar and vertically-resolved aerosol properties

**DOI:** 10.1371/journal.pone.0311990

**Published:** 2024-12-27

**Authors:** Celia Herrero del Barrio, Roberto Román, Ramiro González, Alberto Cazorla, Marcos Herreras-Giralda, Juan Carlos Antuña-Sánchez, Francisco Molero, Francisco Navas-Guzmán, Antonio Serrano, María Ángeles Obregón, Yolanda Sola, Marco Pandolfi, Sara Herrero-Anta, Daniel González-Fernández, Jorge Muñiz-Rosado, David Mateos, Abel Calle, Carlos Toledano, Victoria E. Cachorro, Ángel M. de Frutos

**Affiliations:** 1 Group of Atmospheric Optics (GOA-UVa), Universidad de Valladolid, Valladolid, Spain; 2 Department of Applied Physics, Universidad de Granada, Granada, Spain; 3 Andalusian Institute for Earth System Research, IISTA-CEAMA, Granada, Spain; 4 GRASP-SAS, Remote Sensing Developments, Villeneuve D’Ascq, France; 5 Departamento de Medio Ambiente, Centro de Investigaciones Energéticas, Medioambientales y Tecnológicas (CIEMAT), Madrid, Spain; 6 Departamento de Física, Universidad de Extremadura, Badajoz, Spain; 7 Group of Meteorology, Department of Applied Physics, Faculty of Physics, Universitat de Barcelona, Barcelona, Spain; 8 Institute of Environmental Assessment and Water Research (IDAEA-CSIC), Barcelona, Spain; National College Autonomous, INDIA

## Abstract

This work introduces CAECENET, a new system capable of automatically retrieving columnar and vertically-resolved aerosol properties running the GRASP (Generalized Retrieval of Atmosphere and Surface Properties) algorithm using sun-sky photometer (aerosol optical depth, AOD; and sky radiance measurements) and ceilometer (range corrected signal; RCS) data as input. This method, so called GRASP_*pac*_, is implemented in CAECENET, which assimilates sun-sky photometers data from CÆLIS database and ceilometer data from ICENET database (Iberian Ceilometer Network). CAECENET allows for continuous and near-real-time monitoring of both vertical and columnar aerosol properties. The main characteristics and workflow of CAECENET are explained in detail. This work also explores the potential of CAECENET to monitor and analyze the evolution of transported aerosol events on a regional scale by means of the distribution of CAECENET stations across the Iberian Peninsula. As an example, this paper analyzes, using the CAECENET products, the case of a Saharan dust outbreak that occurred between the 3rd and 5th of October 2022. This was an intense event, with AOD at 440 nm values around 0.5 in Madrid and Valladolid, and reaching 1.55 in Granada. Transport from the Canadian wildfires at the end of June 2023 is also studied. Despite the long-range transport of the smoke particles in this event, measured volume concentrations reached and surpassed 80 μm^3^/cm^3^ in some stations. The results obtained point to the utility of this CAECENET tool for analyzing changes in the height and speed of the event propagation, in the aerosol concentration, and how this affects the optical properties.

## Introduction

The knowledge of the columnar and vertically-resolved atmospheric aerosol properties is crucial for climate studies, as well as for other fields like aviation security and air quality. Moreover, monitoring these properties in near-real-time is important for making early decisions about air quality and pollution, air traffic, and for meeting the demand of information by the general public.

Sun-sky photometers, which measure solar irradiance and sky radiances at different wavelengths, are frequently used to retrieve aerosol properties such as aerosol optical depth (AOD) in the atmospheric column. These automatic instruments are used as reference instrument by some worldwide networks, such as AERONET [AErosol RObotic 10 NETwork; 1], to provide advanced aerosol properties in the atmospheric column in near-real-time (NRT) [2, 3]. A part of the AERONET photometers is managed by the Group of Atmospheric Optics from the University of Valladolid (GOA-UVa). To carry out this management task, the GOA-UVa developed the CÆLIS software tool [4], which receives the photometer data and processes them to generate products like AOD [5].

Measures of AOD and sky radiances from sun-sky photometers are generally used together in inversion codes to retrieve advanced columnar aerosol properties like volume size distribution and the complex aerosol refractive index [6–8], but without vertical resolution. On the other hand, lidars are generally used for the retrieval of vertically-resolved profiles of aerosol properties like aerosol backscattering [9–12] and extinction coefficients [13, 14]. In this sense, some networks like EARLINET (European Aerosol Research Lidar Network) [15] and PollyNET (https://polly.tropos.de) manage multi-wavelength lidar data to provide some aerosol products. However, these lidars are usually expensive and not fully automatic, and there is a scarce supply of this kind of instrument. A cheaper alternative to lidars are ceilometers and single-wavelength lidars, which are fully automatic measuring continuously at one single wavelength. The amount of these low power instruments around the world is much higher. In this sense, there are networks providing ceilometer products in NRT, such as the automatic lidars and ceilometers (ALC) network, which is part of E-PROFILE (https://ceilometer.e-profile.eu), and the Iberian Ceilometer Network [ICENET; 16].

To obtain advanced aerosol properties similar to those with sun-sky photometer but vertically resolved, Román et al. [17], developed the GRASP_*pac*_ method, which combines sun-sky photometer and ceilometer measurements into GRASP (Generalized Retrieval of Atmosphere and Surface Properties) [18, 19] code; retrieving geometric vertical and columnar aerosol properties. This method can be applied to exploit the high amount of data from collocated sun-sky photometers and ceilometers. In fact, the GRASP_*pac*_ method has been already used to retrieve and analyze vertical aerosol properties in some stations like high-mountain locations [20, 21]. However, in these cases, the aerosol products were manually processed, not in NRT, to carry out these specific studies. In this sense, the availability in near-real-time of GRASP_*pac*_ products automatically processed can be useful to exploit synergies between these instruments since it is possible to constantly monitor vertically-resolved aerosol properties and complete the results derived from the aforementioned studies with additional case studies and up-to-date data.

In this framework, the main objective of this work is to develop and establish a system capable of data assimilation from sun-sky photometer and ceilometer networks to apply the GRASP_*pac*_ method and provide NRT vertically resolved and columnar aerosol properties. Databases used to this end are CÆLIS for AOD and sky radiances (sun-sky photometer data) and ICENET for the ceilometer range corrected signal (RCS) data. The combination of both databases (CÆLIS and ICENET) is what gives the developed system its name: CAECENET. The use of CAECENET products for the analysis of regional aerosol events is among the goals of this work in order to test the capability of this new tool.

This paper is structured as follows: first, the CAECENET system is presented, its main characteristics, structure and workflow; the results section presents the detailed analysis of two aerosol events over the Iberian Peninsula using CAECENET products; finally, the main conclusions of this work are summarised.

## Instruments and data processing

CAECENET is a system that has implemented the automatic application of the GRASP_*pac*_ method using sun-sky photometer and ceilometer data. All these data are obtained directly from CÆLIS and ICENET databases, respectively, which are explained in the next subsections.

### CÆLIS and photometer data

The CE318 sun-sky photometer (*Cimel Electronique*) is the main instrument of AERONET. It is basically formed by a head with one Silicon and one InGaAs sensor with a filter wheel and a collimator tube, that measures at several wavelengths (generally 340, 380, 440, 500, 675, 870, 940, 1020 and 1640 nm) the Sun direct irradiance and the sky radiance at different sky angles thanks to a 2 axis robot. A recent model (CE318-T) also allows lunar irradiance measurements to derive the AOD at night-time [22–26]. This new model is also capable to perform the sky radiance measurements in three different geometries: Almucantar, where the zenith angle is equal to the solar zenith angle and the measurements are done for different sky azimuth angles; Principal Plane, where the azimuth angle is equal to the solar azimuth angle and the measurements are carried out for different sky zenith angles; and Hybrid, which is a combination of Almucantar and Principal Plane. More information about these sky radiance scenarios can be found in Sinyuk et al., 2020 [3].

CÆLIS is a web tool developed by the GOA-UVa with the main goal of managing the calibration and quality control of the AERONET photometers that the calibration center at Valladolid is in charge of. CÆLIS, based on a client-server architecture, is composed of three main modules: 1) a relational database to store the raw photometer measurements of each instrument, but also the ancillary data needed for the retrieved products, such as optical depth from different gases or climatology values of the Bidirection Reflectance Distribution Function (BRDF); 2) a web interface that allows the monitoring of the performance of all different instruments; and 3) a processing chain that provides products, such as AOD and sky radiances, in NRT. These three modules are explained in detail in Fuertes et al., 2018 [4].

The processing chain of CÆLIS starts when a raw data file from a photometer is received in the server, activating a set of triggers in the NRT module. The NRT module processes the data to retrieve products but also assimilates the needed ancillary data, which are used to compute the AOD and sky radiances [5]. There are two levels of data for AOD, level 1.0 for all measurements even contaminated by clouds, and level 1.5, where the data are cloud screened following the criteria explained in González et al., 2020 [5] based on Giles et al., 2019 [2].

### ICENET and ceilometer data

The Iberian Ceilometer Network (ICENET) is an initiative of the Atmospheric Physics Group of the University of Granada for the coordination and validation of ceilometer measurements at a regional scale. The main goal of ICENET is obtaining reliable vertically resolved aerosol properties in NRT [16].

**Fig 1:**
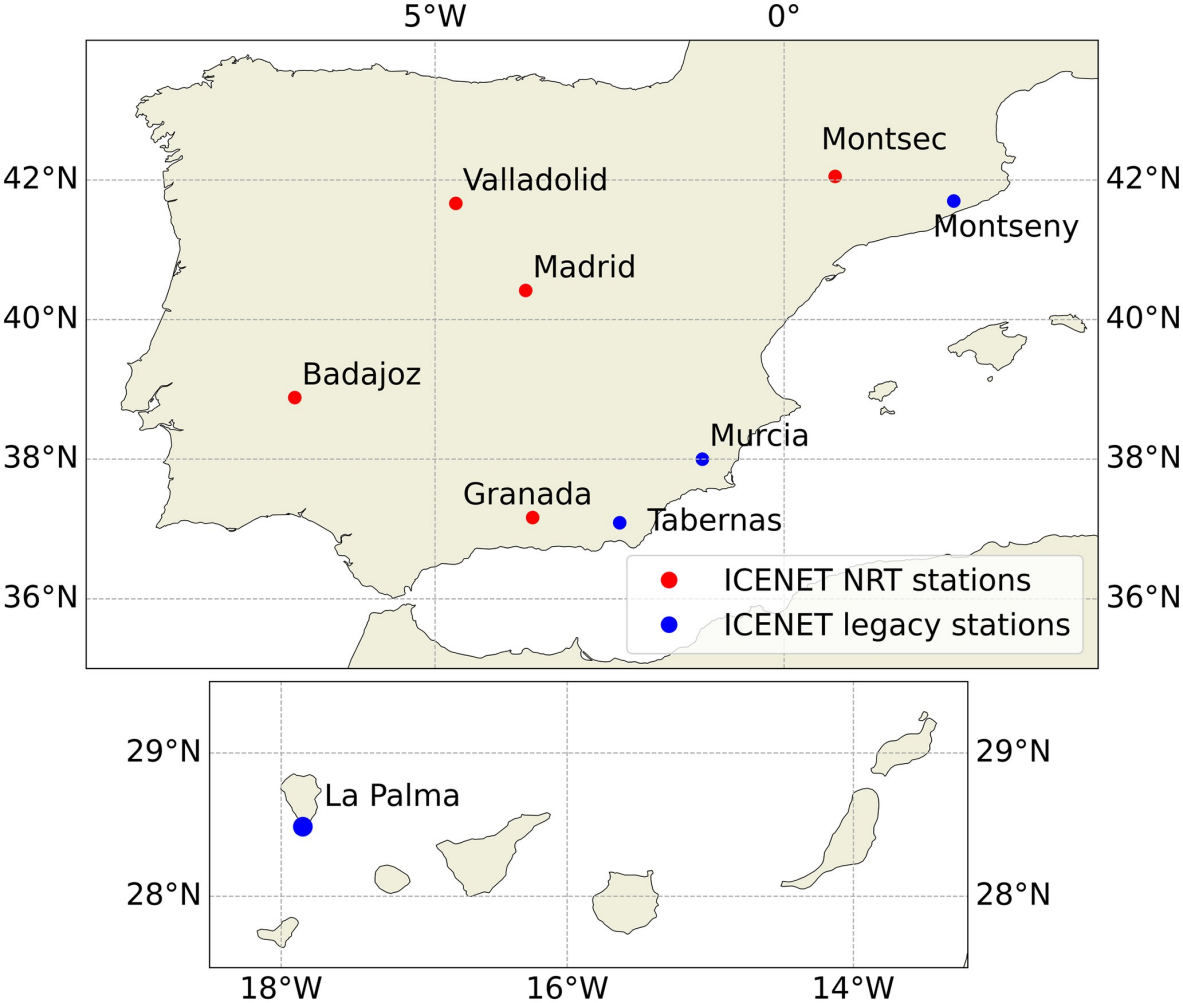
Map of the Iberian Peninsula, including the Canary and Balearic Islands, showing the location of ICENET stations. Red dots represent the ceilometers operating in near-real-time conditions while blue dots correspond to legacy stations providing data in the past or intermittently. Figure obtained using the Python Cartopy library.

Currently, five stations are operating in NRT and another four stations had provided data in the past or intermittently. Fig 1 shows the spatial distribution of the stations.

The main instrument in ICENET is the Lufft CHM15k-Nimbus ceilometer. This ceilometer operates with a pulsed Nd:YAG laser emitting at 1064 nm and has vertical resolution of 15 m. The vertical range expands from 15 to 15360 m. The instrument has its own overlap correction and for all instruments in ICENET, the overlap function provided by the manufacturer shows an overlap over 90% between 555 and 885 m a.g.l. Most stations also have a collocated sun-sky photometer from AERONET and AOD information is used as ancillary information for the calibration and inversion modules explained bellow.

ICENET operates with several modules:

- The acquisition module obtains data from the ceilometers and organizes them in the corresponding dataset. AERONET data is also obtained for those stations with collocated sun-sky photometer.
- The calibration module calibrates the RCS following the procedure described by Cazorla et al. [16] and provides attenuated backscatter coefficient.
- The inversion module provides backscattering coefficient and extinction coefficient applying the Klett-Fernald inversion [9, 10] to the RCS.
- The CAECENET module prepares the RCS signal from the ceilometers in the adequate format for the GRASP_*pac*_ retrieval and sends it to CAECENET (see next section).

All these modules can operate in NRT for connected stations that provide ceilometer data. For non-connected stations or for case studies, manual operation is also possible.

### GRASP_*pac*_ retrieval

The GRASP algorithm is a versatile inversion code capable to retrieve atmospheric and surface properties, among others, using different sets of observations. This code is based on multi-term least square method [6] but its flexibility allows the stand-alone inversion of the measurements of different instruments like: satellite instruments [27, 28], sun-sky photometers [29–31], lidars [32], sky cameras [33], polar nephelometers [34] or zenithal radiometers [35], among others; but also the joint inversion of combined measurements from different instruments, such as sun-sky photometers plus lidars [36–38] or plus sky cameras [25, 39].

One of the inversion strategies developed for GRASP is the so called GRASP_*pac*_ (*pac* is the acronym of *photometer and ceilometer*), which combines AOD and sky radiance measurements from sun-sky photometer with RCS data from ceilometer. The GRASP_*pac*_ method uses as input the measurements of AOD and sky radiance at 440, 675, 870 and 1020 nm from photometers, and the measured RCS at 1064 nm from ceilometers, with the possibility of selecting other wavelengths. Each GRASP_*pac*_ retrieval is centered in time for the sky radiance measurements, which can be performed under Almucantar or Hybrid scenarios. These sky radiances are previously filtered by clouds assuming that measurements performed under symmetric scattering angles must show differences below 20% between both branches. A GRASP_*pac*_ retrieval is not performed if there are not more than 10 sky radiance values per wavelength and at least one measurement in four different scattering angle ranges (3.2° to 6°, 6° to 30°, 30° to 80° and 80° to 180°). The AOD (cloud-screened) closest in time to the sky radiance values within ± 16 min are used in the process. Regarding RCS, it is firstly cloud-screened and averaged over a ± 15 min window centered around the sky radiance time. The time averaged RCS undergoes vertical smoothing through a moving average with a window of ± 105 m to mitigate noise. Subsequently, it is normalized across 60 log-spaced bins at various heights, following the approach outlined by Lopatin et al., 2013 [36]; the height range for normalization spans from a minimum of 250 m a.g.l. to a dynamic maximum which is usually about 7000 m a.g.l. The determination of this dynamic maximum and the normalization process is well described by Román et al., 2018 [17]. In addition, GRASP_*pac*_ needs to introduce in GRASP the BRDF, which is modeled by the three parameters of the Li–Ross model [40, 41].

Regarding the inversion strategy, GRASP_*pac*_ method approaches the aerosol volume size distribution to a log-space 22 triangle bins distribution with radius from 0.05 to 15 *μ*m without discerning between fine and coarse modes intensive properties; the real and imaginary parts of refractive index are considered as wavelength dependent; and the sphericity, which quantifies the portion of spherical particles and spheroids, is also retrieved. The intensive aerosol properties, like complex refractive index or the shape of volume size distribution, are assumed invariant with height. However, extensive aerosol properties, like aerosol volume concentration (VC) or extinction coefficients, are assumed as height-dependent. The variations of volume size distribution with radius, complex refractive index with wavelength, and aerosol vertical distribution with height, are constrained to be smooth. An *a priori* constraint [19] is also used for the smoothness of the volume size distribution in GRASP_*pac*_ method.

As a result, GRASP provides different columnar and vertically-resolved aerosol products. In column: one value of sphericity; the volume size distribution (normalized by particle volume concentration) at 22 radius bins and the total aerosol volume concentration; and five values (one per wavelength) of the real and imaginary parts of the refractive index, the phase function, and other derived products like lidar ratio, single scattering albedo (SSA) and AOD. Regarding vertical properties, GRASP provides the aerosol normalized vertical profile (AVP) at the chosen 60 heights and at ground level, which AVP value is assumed equal to the obtained at the closest height level. The AVP data represents the normalized vertical aerosol concentration, which is used to derive some aerosol extensive properties like profiles of aerosol volume concentration and extinction coefficient, multiplying AVP by total columnar volume concentration and AOD, respectively. Profiles of aerosol scattering coefficient are derived by multiplying extinction by SSA; absorption coefficient profiles as the substraction of extinction minus scattering coefficients; and profiles of backscattering coefficient multiplying the extinction coefficient by lidar ratio.

Furthermore, GRASP counts with its own methodology to calculate the columnar effective radius, volume concentration, and AOD for fine and coarse modes separately. Subsequently, vertical profiles of extinction coefficient and other properties can be computed for the five wavelengths and for the fine and coarse modes separately. However, the shape of these profiles remains equal across all wavelengths and for both modes, following the shape of AVP, since the RCS is only available for one wavelength, preventing the differentiation between fine and coarse particles. Consequently, AVP cannot be determined separately for fine and coarse modes, as observed in multi-wavelength lidar retrievals. As a result, intensive properties such as the Angström Exponent (AE) and the fine/coarse fraction do not exhibit variations with height, even when the profiles of different properties like extinction coefficient are available for different wavelengths and modes.

### CAECENET structure and workflow

As mentioned, CAECENET is a system that has been created to run automatically the GRASP_*pac*_ method using the sun-sky photometer and ceilometer data from CÆLIS and ICENET as input [42]. CAECENET has been developed as a system with three different main modules: NRT module, database and viewer.

**Fig 2:**
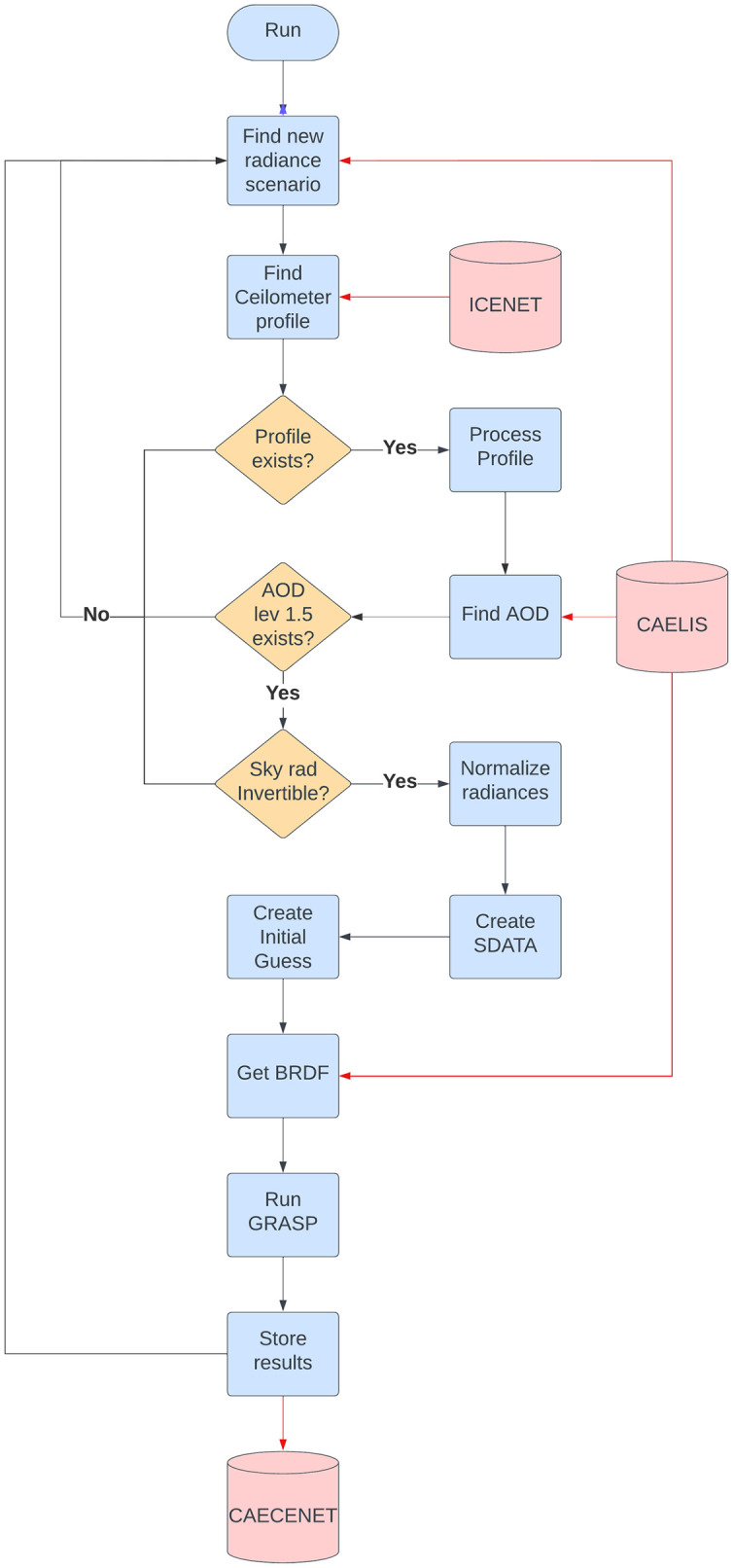
Workflow of the CAECENET algorithm.

Regarding the NRT module, it is responsible for obtaining and merging in one input file the AOD and sky radiances from CÆLIS and the RCS vertical profiles from ICENET and run GRASP code with the GRASP_*pac*_ configuration. The workflow of this CAECENET task can be shown in Fig 2. The blue lines on this diagram describe the workflow of the CAECENET main algorithm and the red lines all those sub-modules of the algorithm where data from different databases (CÆLIS and ICENET) are consulted. First of all CAECENET looks for new sky radiance observations in CÆLIS database. If there is a new sky radiance measurement (Almucantar or Hybrid), then CAECENET looks for RCS vertical profiles provided by ICENET. If the RCS data passes the quality-assurance and cloud-screening criteria, then the RCS data is averaged to 60 log-spaced points and CAECENET looks for the AOD level 1.5 (cloud-screened) in CÆLIS database. If the AOD is available, the sky radiance measurements are checked, and if they pass the mentioned cloud-screening and quality assurance criteria (i.e., they are invertible), then these radiances are normalized following the GRASP format. This normalization takes into account the measured spectral response of each filter in each sun-sky photometer convoluted by the extraterrestrial spectrum *2000 ASTM Standard Extraterrestrial Spectrum Reference E-490–00* (http://rredc.nrel.gov/solar/spectra/am0).

All these input values are written for each scenario in a SDATA (Sensor Data) file, following the working methodology of GRASP code. This algorithm needs, to make a retrieval, a SDATA file with the input data, but also a SETTINGS file where the inversion strategy and approaches are included. Then, once the SDATA is obtained, GRASP (version 1.0.1) is run. For this task, CAECENET also modifies the SETTINGS introducing the BRDF values obtained from CÆLIS (climatology tables of V005 Collection MCD43C1 product: V005 MODIS Terra+Aqua BRDF/Albedo 16-Day L3 0.05Deg CMG) and the initial guess values of the size distribution for the chosen scenario. This initial guess depends on the AOD at 440 nm value, as explained in previous works [17].

If there is no cloud-free data of RCS, or AOD level 1.5, or cloud-free sky radiances, then GRASP_*pac*_ is not run and CAECENET will look for another one. After each retrieval, the output is stored in a file. The NRT module also allows the manager of the system to run or stop NRT for all CAECENET sites and also can be used to reprocess data from the past.

The main results of the GRASP_*pac*_ output files are also stored into the CAECENET database by the database module. CAECENET has been built based on a relational database to make all the information available for different users and provide a search system. The schema of the used database is simple and scalable, since it stores the output of the GRASP_*pac*_ retrievals, explained above, discerning four categories: vertical profile properties, optical columnar properties (which also includes asymmetry parameter derived from phase function), microphysical columnar properties and internal status. The data structure of those four categories is different, taking into account the peculiarities of the outputs, as has been explained in the previous subsection.

Finally, to visualize the data produced by CAECENET exits the third main module, a desktop application that acts as a viewer. This viewer allows the users to visualize the data at any station [42]. The output errors are provided as well, obtaining the ± error interval for each retrieved parameter [43].

## Results

### Regional dust aerosol event

**Fig 3:**
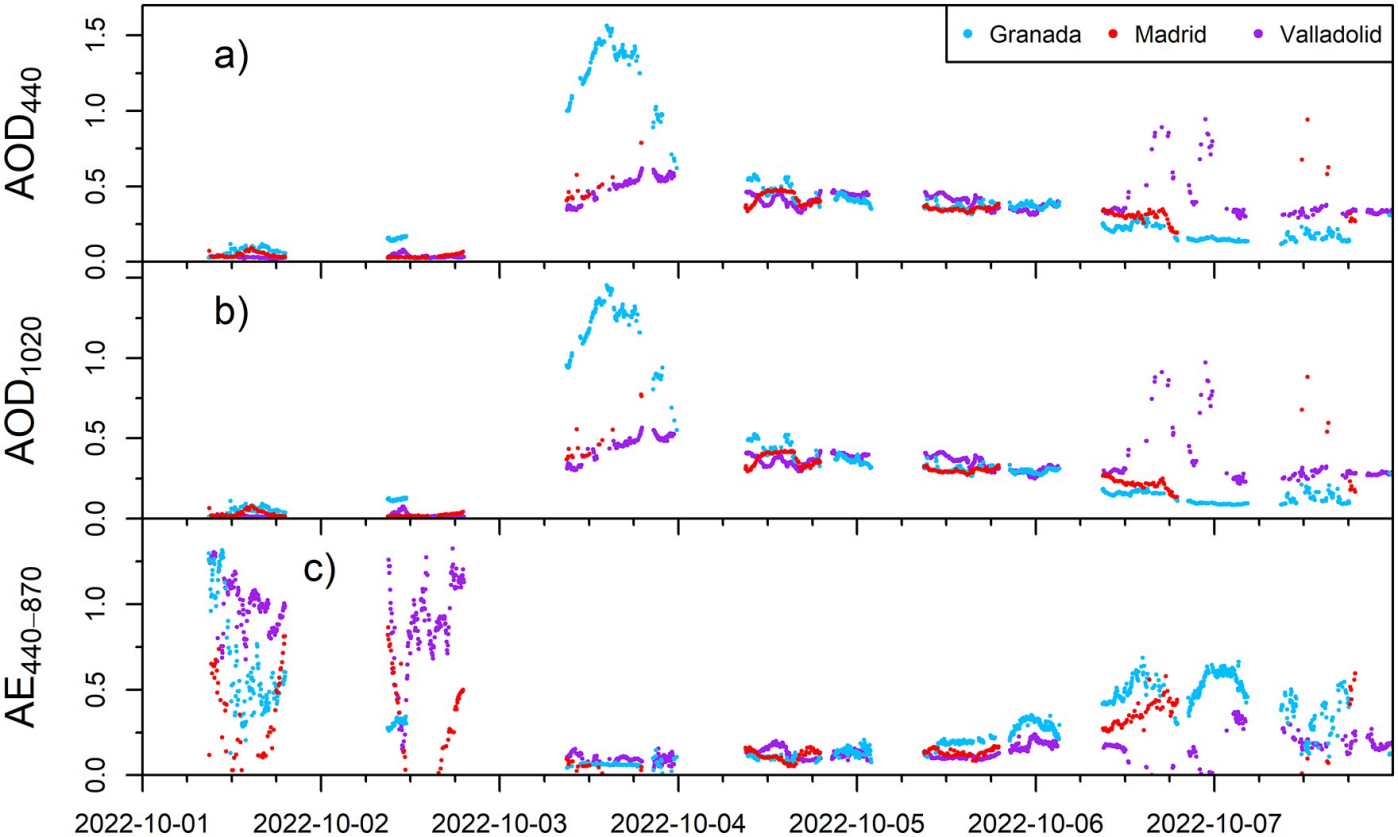
AOD values at a) 440 nm, b) 1020 nm and c) Angström Exponent (AE), provided by CÆLIS (level 1.5), for Granada, Madrid and Valladolid stations from October 1st to October 7th, 2022.

A high aerosol load event that occurred in the Iberian Peninsula in early October 2022 has been identified and regionally analyzed using CAECENET tool. Data for these days is available for the Granada, Madrid, and Valladolid stations. Fig 3 shows the time series for AOD level 1.5 measurements at 440 and 1020 nm and AE (calculated with all the channels between 440 and 870 nm) provided by CÆLIS. On October 3rd there was a strong increase in the AOD values, compared to the previous days (< 0.1 at all sites), reaching a maximum value of 1.55 at 440 nm in Granada around 13:00 UTC. In Valladolid and Madrid sites, the AOD values on that day were about 0.5, above the typical values for these stations in central Iberian Peninsula [44]. Specifically, the maximum AOD value at 440 nm in Valladolid and Madrid were 0.62 and 0.77, respectively, both around 17:00 UTC. The AOD values at 1020 nm (Fig 3b) were very similar to those at 440 nm for the three stations on 3rd October, implying low spectral dependence on AOD and therefore AE values below 0.2 as observed in Fig 3c for the three locations, indicating that the aerosol particles were mainly coarse. This is a first indicator of a Saharan desert dust outbreak, which fits with other reported events detected in the region [e.g., 45–49]. The AOD values were still about 0.5 with low AE values during 4th and 5th October for the three locations, pointing out the remaining presence of coarse particles. The AE started to increase at the beginning of the night from 5th to 6th October, as can be seen for Valladolid and Granada where a sun-sky-moon photometer (CE318-T) was available. Finally, the AOD on 6th October decreased, particularly for Madrid and Granada, with higher AE values than the previous days, which indicates the end of the coarse-mode dominated event. High AOD values in Valladolid are observed during October 6th but they were most likely caused by the presence of high-altitude clouds such as cirrus (which has been confirmed in images from a sky camera installed at Valladolid).

**Fig 4:**
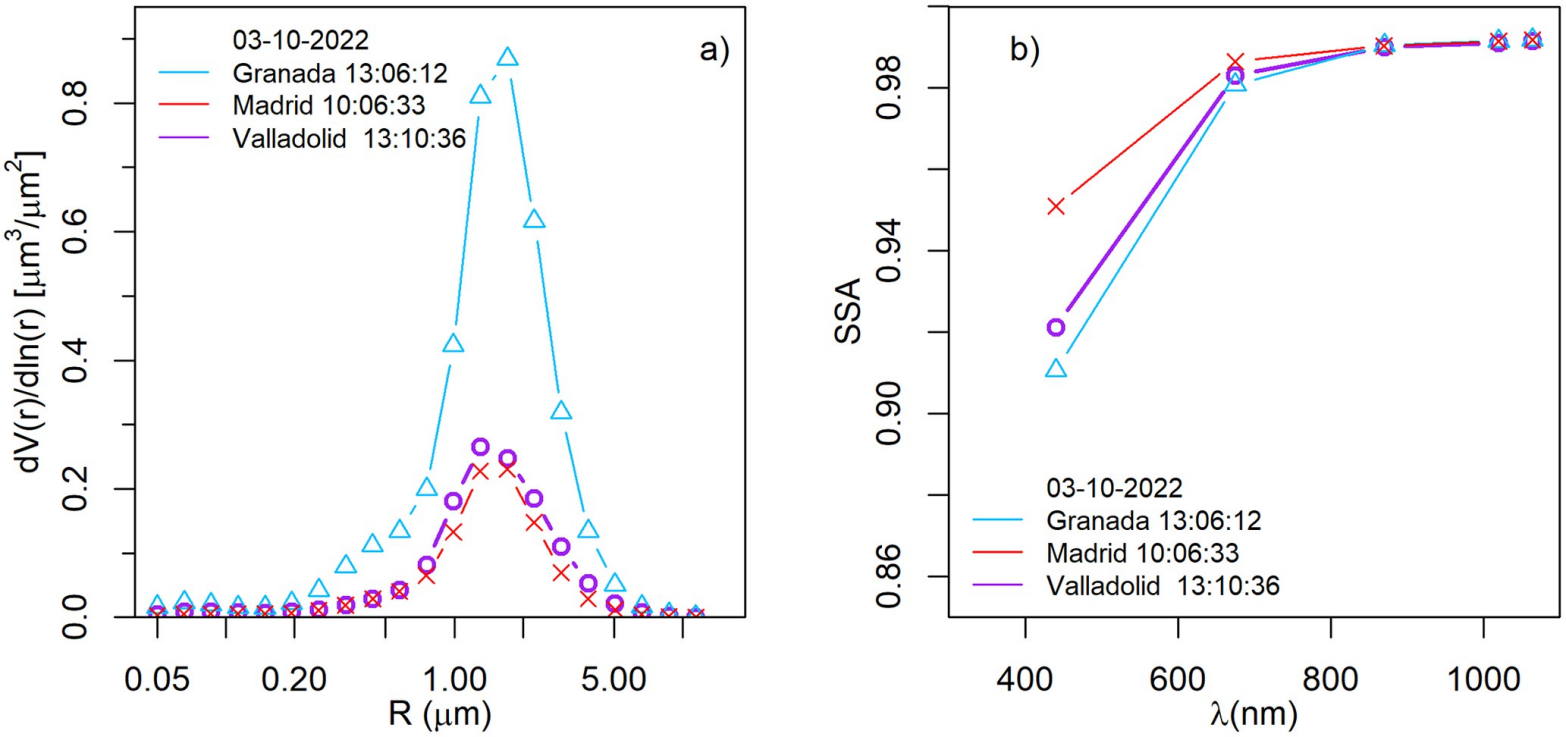
Volume size distribution a) and single scattering albedo (SSA) b) obtained with *GRASP*_*pac*_ and provided by CAECENET for the maximum aerosol load reached on 3rd October at Granada, Madrid, and Valladolid.

Fig 4 shows the aerosol volume size distribution (panel a) and the SSA (panel b) for the CAECENET retrieval with the highest AOD value on 3rd October. The highest volume concentration, with values of approximately 0.86 μm^3^/μm^2^ in Granada and about 0.26 μm^3^/μm^2^ and 0.23 μm^3^/μm^2^ at Valladolid and Madrid, respectively, appears for a particle radius about 2.25 μm in the three stations. It reveals a clear presence of coarse mode, while fine mode is negligible. The SSA, shown in Fig 4b), is above 0.98 for 675 nm to 1020 nm, pointing out that the light absorption of the present aerosol was very low at these wavelengths for the three sites. However, the absorption is higher at 440 nm, especially for Valladolid and Granada where SSA values were about 0.92 and 0.91 at this wavelength. The observed characteristic of size distribution (coarse particles) and SSA values (very low absorption except for the shortest wavelengths) fit with those expected for Saharan dust aerosols [49–51].

**Fig 5:**
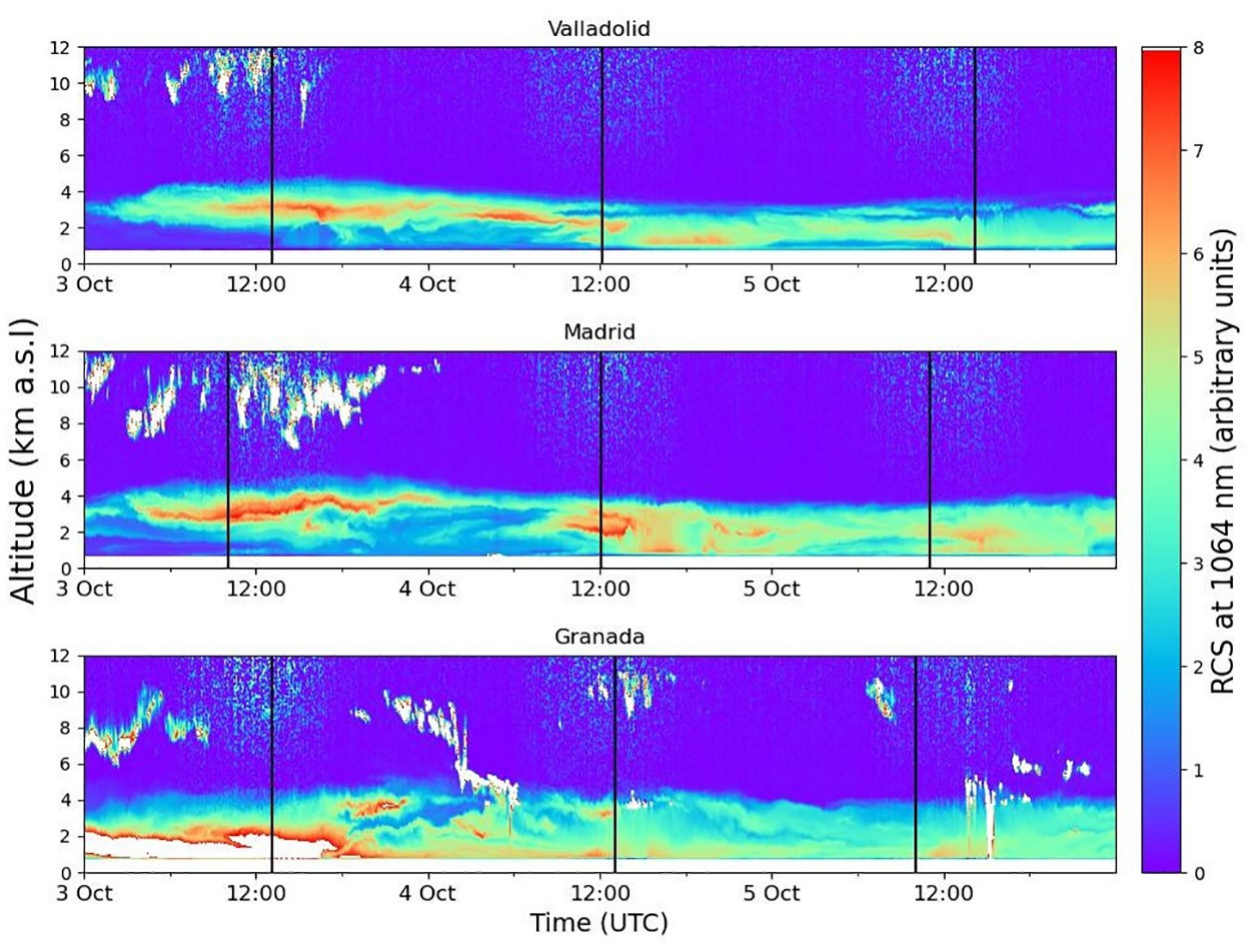
Ceilometer time series of range corrected signal representing the evolution of the dust outbreak between 3rd and 5th October 2022 at the stations of Granada (680 m), Madrid (669 m) and Valladolid (705 m). Black vertical lines indicate the time of the profiles in Fig 6 for each station.

The time series of the ceilometer RCS at each station is shown in Fig 5 to qualitatively determine the altitude reached by the aerosol particles during this event. On October 3rd in Granada, a substantial aerosol layer is observed at a very low altitude, ranging from the surface up to 2 km a.s.l. and persisted until the afternoon. The aerosol load in this layer seems to be high (the values are above the chosen maximum value in the colorbar legend) which is in line with AOD values about 1.55 mentioned above; this layer disappeared at late afternoon when the AOD started to decrease and other aerosol layer appeared between 2 and 4 km a.s.l. A higher aerosol layer, between 2 and 3 km a.s.l. arrived in Madrid and Valladolid at the beginning of the same day. These layers persisted throughout the day and became thinner at the end of the day. Fig 5 also shows a high presence of high-altitude clouds in Madrid on 3rd October, which are the main responsible for the scarcity of cloud-screened AOD data on that day. On October 4th, there is a slight decrease in aerosol load and a progressive reduction in altitude of the layer. Two decoupled aerosol layers can be observed around midday in Madrid and Valladolid, later the aerosol appears to be mixed within the planetary boundary layer. By October 5th, it is observed that the aerosol load continues to decrease, with the layer remaining below 4 km a.s.l., with the main part near the surface. In addition, some rain precipitation occurred between 14:00 and 15:00 UTC in Granada that could contribute to the wet deposition of aerosol particles. However the aerosol layer still persists after the precipitation as it can be observed in the temporal series of the RCS (Fig 5).

**Fig 6:**
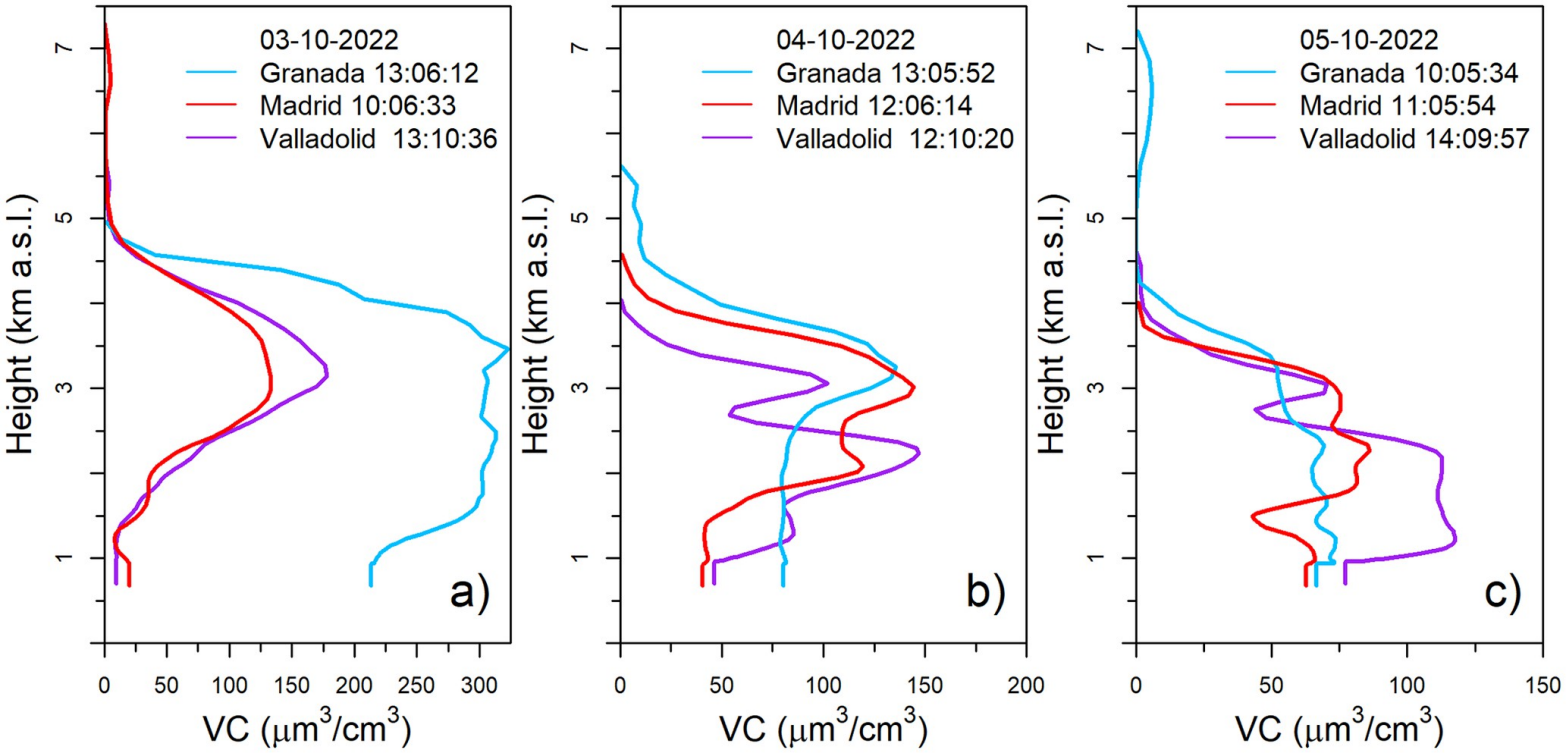
Volume concentration (VC) vertical profiles (altitudes from 0 to 7.5 km above sea level) at the studied stations. In panel a), the values are shown for October 3rd, in panel b) for October 4th, and in panel c) for October 5th 2022, at the hours (UTC) indicated in the legend for each station.

For a more quantitative characterization, Fig 6 presents vertical volume concentration profiles, obtained from CAECENET, between October 3rd and 5th. The chosen profiles correspond to the available retrievals with the highest AOD value on each day, which are expected to exhibit the highest volume concentrations. On October 3rd, there was a strong aerosol volume concentration in Granada between 1.5 and 4 km a.s.l. with values between 250 and 325 μm^3^/cm^3^. The volume concentration in Madrid and Valladolid presented different behavior than in Granada, which could be attributed to the higher distance and the orography between southern and central Spain. The VC profiles in Madrid and Valladolid were similar, being mainly distributed in a 2 km width layer centered around 3 km a.s.l. The maximum values were about 130 and 180 μm^3^/cm^3^ in Madrid and Valladolid, respectively. The split of the mentioned aerosol layer (see Fig 5) in two decoupled aerosol layers can be observed the next day (October 4th) centred at 2 and 3 km a.s.l. with the highest value close to 150 μm^3^/cm^3^ in both stations. This day, a layer around 3 km a.s.l. also appeared at Granada with similar VC values than in Madrid. Finally, on 5th October, the layer structures were no so evident and the peaks of VC appeared in lower heights and with lower values, (below 125 μm^3^/cm^3^, 90 μm^3^/cm^3^ and 75 μm^3^/cm^3^ for Valladolid, Madrid and Granada, respectively).

The vertical profiles of the extinction, scattering, absorption, and backscattering coefficients are also available from CAECENET for the days of the event (shown in the S1 Fig). The absorption coefficient values were low, as expected from desert dust particles, for 440 nm wavelength, being lower than 10 Mm^−1^ for Madrid, less than 30 Mm^−1^ for Valladolid and 45 Mm^−1^ for Granada. The scattering coefficient at 440 nm, reached values up to 200 Mm^−1^, 240 Mm^−1^ and 430 Mm^−1^ for Madrid, Valladolid and Granada, respectively. The backscattering coefficient presented values lower than 10 Mm^−1^sr^−1^ at 440 nm for all three stations and, finally, the extinction coefficient presented values slightly exceeding the scattering coefficient, at 440 nm. The maximum value at Valladolid was 265 Mm^−1^, 205 Mm^−1^ for Madrid and 469 Mm^−1^ at Granada.

**Fig 7:**
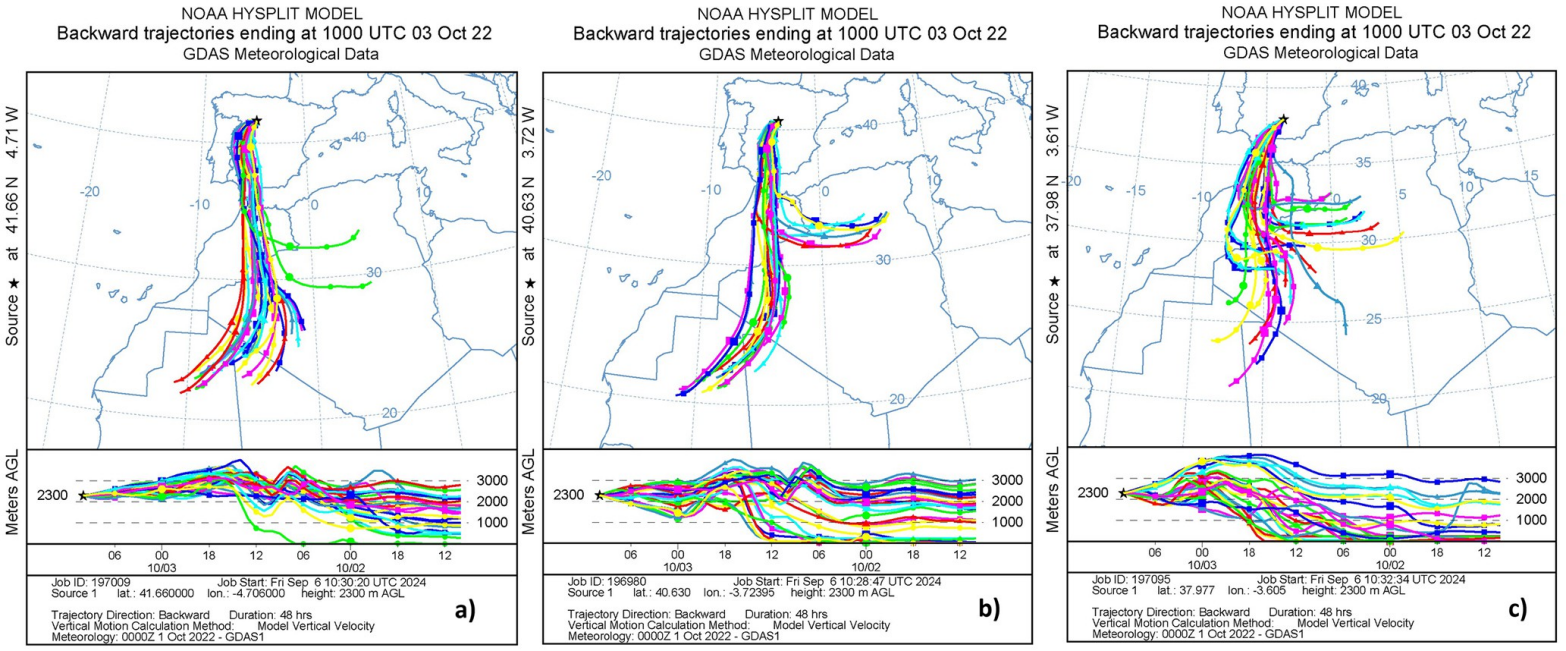
Back-trajectories from a) Valladolid, b) Madrid and c) Granada stations, calculated by HYSPLIT, at altitude level of 3 km on the 3rd October 10:00 UTC.

All the results point out that the aerosol type is Saharan dust. To corroborate the origin source of these particles, the HYSPLIT [Hybrid Single-Particle Lagrangian Integrated Trajectory, 52] backward trajectory calculation model has been used. It has been run using the Global Data Assimilation System (GDAS) meteorological database and computing the back-trajectories for 48 hours prior to the arrival of air masses for the three stations at altitudes of 3000 m a.s.l. (chosen based on the CAECENET results of Fig 6) on October 3rd at 10:00 UTC. The obtained back-trajectories are shown in Fig 7, revealing that all back-trajectories come from the northern part of Western Sahara. The air mass altitude was low on October 1st and 2nd and was elevated during its transport to the Iberian Peninsula. The majority of trajectories originate from the same region. Only a few emerge from further to the east, more frequently in Granada than in Madrid and Valladolid. This could explain the differences in the volume concentration profiles.

Once the aerosol origin has been identified, the NCEP/DOE meteorological reanalysis has been analyzed to characterize the synoptic situation. A surface-level low-pressure system began to form over the Sahara region on September 30th (not shown). This led to pump Saharan dust into the atmosphere. Additionally, on October 1st, a high-pressure system started to develop at 700 hPa (about 3 km.) This anticyclone was responsible for the transport of the dust towards the Iberian Peninsula. This scenario is referred to as NAH-A (North African High Located at Upper Levels) according to the classification of Escudero et al., 2005 [53], and it is the most common pattern in the Peninsula between May and October [48]. All these results confirm that the analyzed event was formed by Saharan mineral dust particles and explain the origin and transport mechanisms to reach the Iberian Peninsula at the observed heights in the CAECENET data.

### Wildfire smoke event

In summer 2023 Canada suffered several forest fires that burned an estimated 18.4 million hectares [54]. The intensity of the fires during June in the state of Quebec, resulted in the formation of a plume, generated by the extreme heat and convection triggered by the burning vegetation [55]. Due to the predominant westerly winds prevalent at midlatitudes and the robust convection observed over Canada, which can lift smoke to high altitudes, even far above the planetary boundary layer, this plume could potentially travel long distances in accordance with local atmospheric circulation patterns [56, 57]. CAECENET network has enabled to monitor this long-range transport smoke event to the Iberian Peninsula.

**Fig 8:**
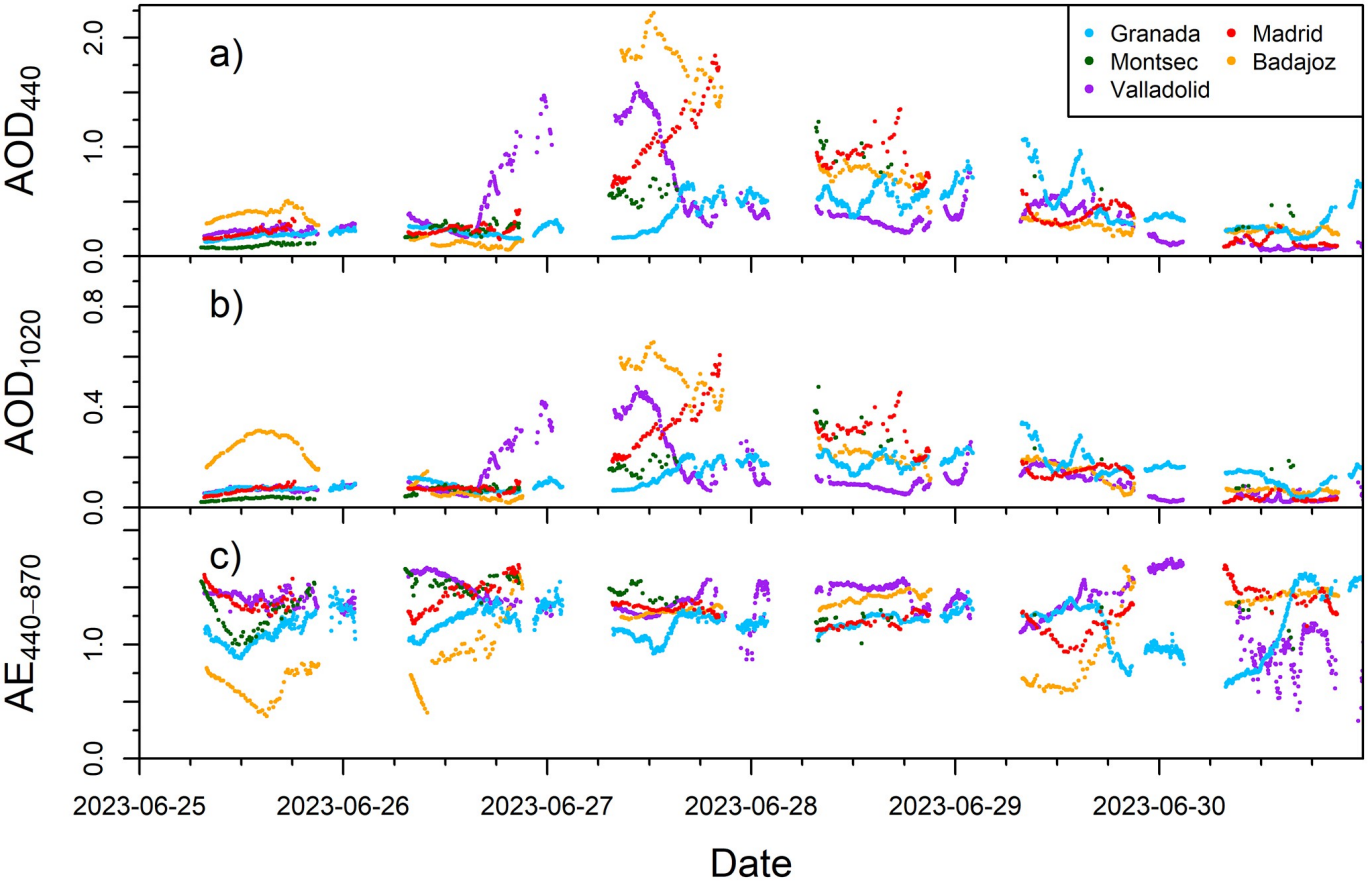
AOD values at a) 440 nm, b) 1020 nm and c) Angström Exponent (AE), provided by CÆLIS (level 1.5), for Granada, Badajoz, Madrid, Montsec and Valladolid stations from June 25th to 30th, 2023.

Fig 8 shows the AOD and AE for the end of June 2023 at several CAECENET stations in the Iberian Peninsula. First, before noon on 26th, the AOD at 440 nm values at all stations were close to 0.2, not significantly above the average for those stations. Subsequently, a sharp increase was observed in Valladolid, reaching an AOD value at 440 nm of 1.47. The following day, the values in Valladolid values remain high and an increase is early observed in Badajoz (AOD at 440 nm reaching 2.22), but also in Madrid (AOD at 440 nm up to 1.83) and Montsec (AOD at 440 nm about 0.56); this increase appeared in Granada at midday reaching AOD values at 440 nm close to 0.61. This increase in AOD was associated with AE values around 1.27, corresponding to predominant fine particles but with larger fine mode median size, due to coagulation and condensation processes during the large transport [58, 59]. On June 28th, extreme values decrease in Valladolid, Madrid, and Badajoz, but high AOD values still persist, indicative of a high-turbidity event across all stations, notably prominent in Montsec due to its elevated altitude and generally clean environment. Finally, on June 29th, lower AOD values began to recover at all stations, the AE showing different values among the stations.

**Fig 9:**
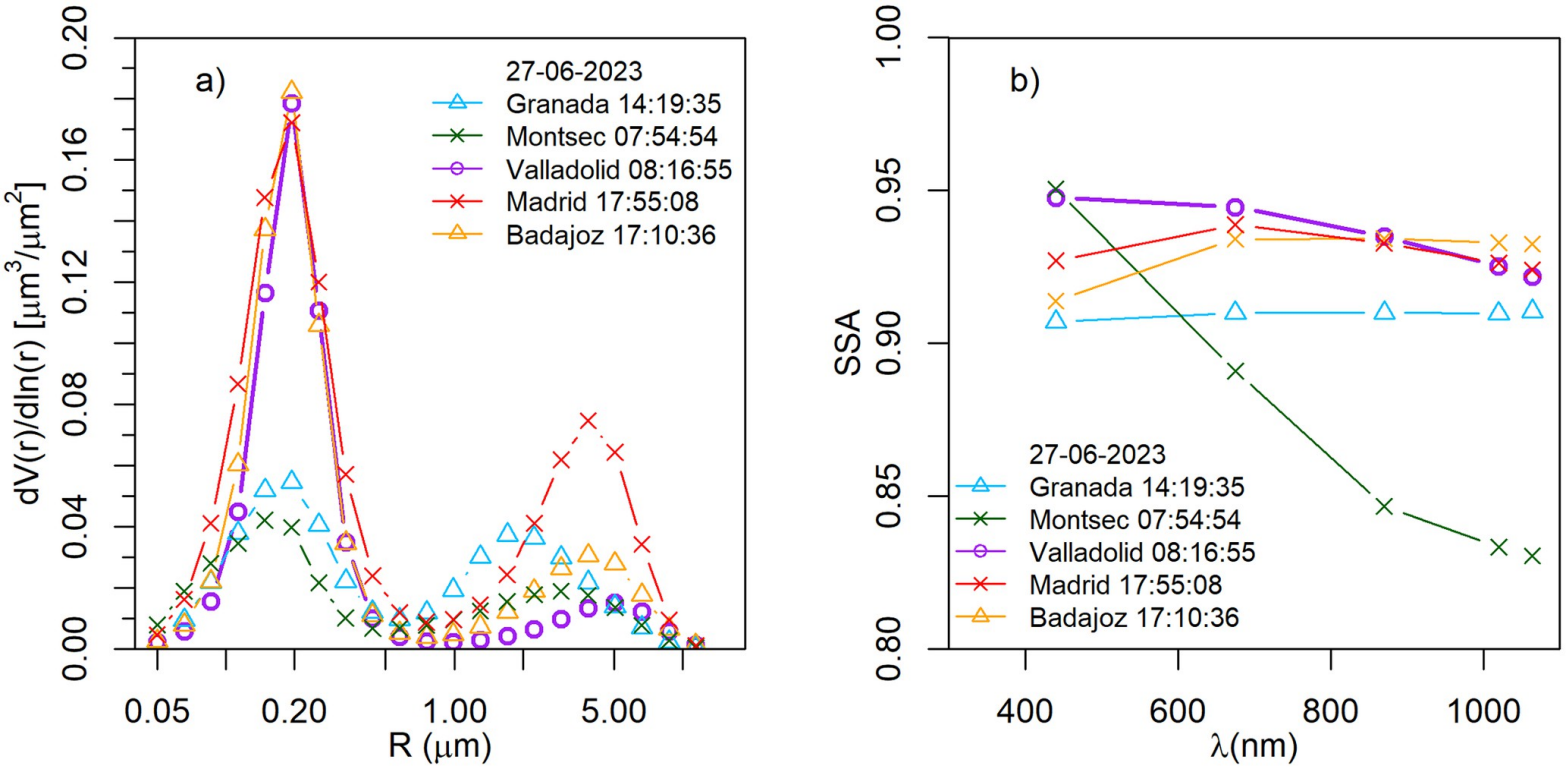
Size distribution a) and SSA b) obtained with *GRASP*_*pac*_ and provided by CAECENET for the 27th of June, at UTC hours shown for each station.

Regarding the CAECENET columnar aerosol properties, the volume size distribution and the SSA values are shown in Fig 9 for June 27th. Volume size distribution showed a fine predominant mode, with a high peak in Valladolid, Madrid, and Badajoz, reaching almost 0.2 μm^3^/μm^2^, centered around 0.2 μm. The retrieved fine modal radius for this day was 0.23 μm on average for all the stations, a typical value for aged smoke [60, 61]. In Montsec and Granada, the volume concentration for this day was lower because, as AOD has revealed, the highest aerosol load reached these stations one and two days after. The SSA values for Madrid and Badajoz exhibit a very similar trend, with a slight peak at 675 nm. In the case of Valladolid, a SSA decrease with the wavelength is observed, starting from 0.95 at 440 nm. In Granada values seem constant around 0.91. These values are associated with smoke particles [50], and so, the AE values seen in Fig 8, therefore, the hypothesis that they may have been transported from the wildfires in Canada seems plausible.

**Fig 10:**
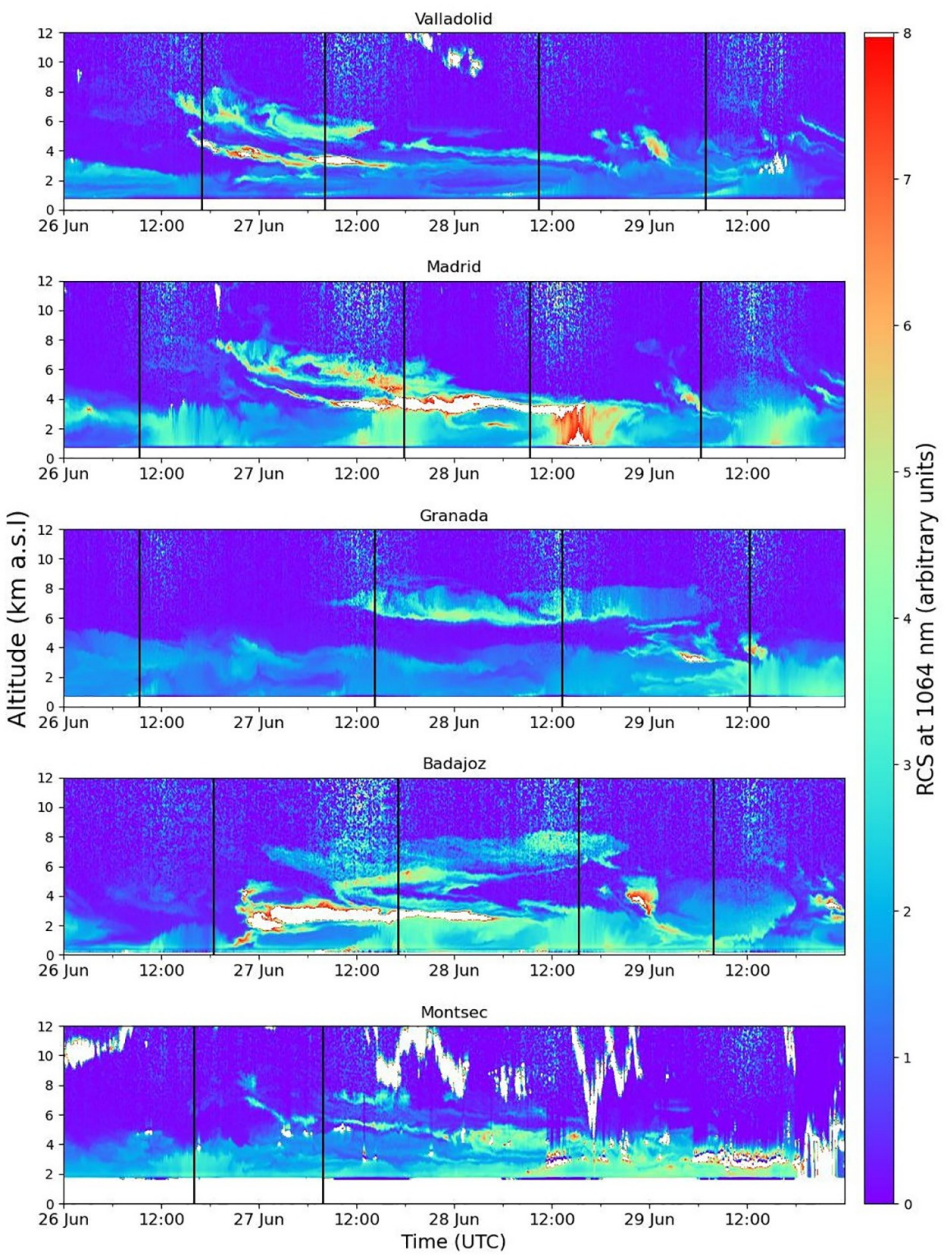
Ceilometer time series of range corrected signal representing the evolution of the event between 26th and 29th June 2023 at the stations of Valladolid (705 m), Madrid (669 m), Granada (680 m), Badajoz (186 m) and Montsec (1574 m). Black vertical lines indicate the time of the profiles in Fig 11 for each station.

It is possible to analyze the information in terms of altitude and track the evolution of the event through the ceilometer data from each station; in order to do so, the time series of the ceilometer RCS is plotted in Fig 10. It can be observed that an aerosol plume reached first Valladolid in the afternoon, followed by Madrid in the evening, and Badajoz and Montsec on late June 26th. The aerosol entered in two main thin layers around 5 and 8 km a.s.l., after that altitude started going quickly down to 3 and 5 km a.s.l. on June 27th. In Granada the aerosol layer arrived around midday on June 27th. The following day it is noticeable the progression of the aerosol layer, situated slightly higher than in the other stations, above 6 km a.g.l., and it is also gradually dissipating. This event occurred in Montsec in the presence of clouds, which explain the scarcity of AOD data in this station. It is possible to observe a thin layer entering around 4 km a.g.l. on June 27th, and on June 28th, it is also observed at a slightly lower altitude before disappearing.

**Fig 11:**
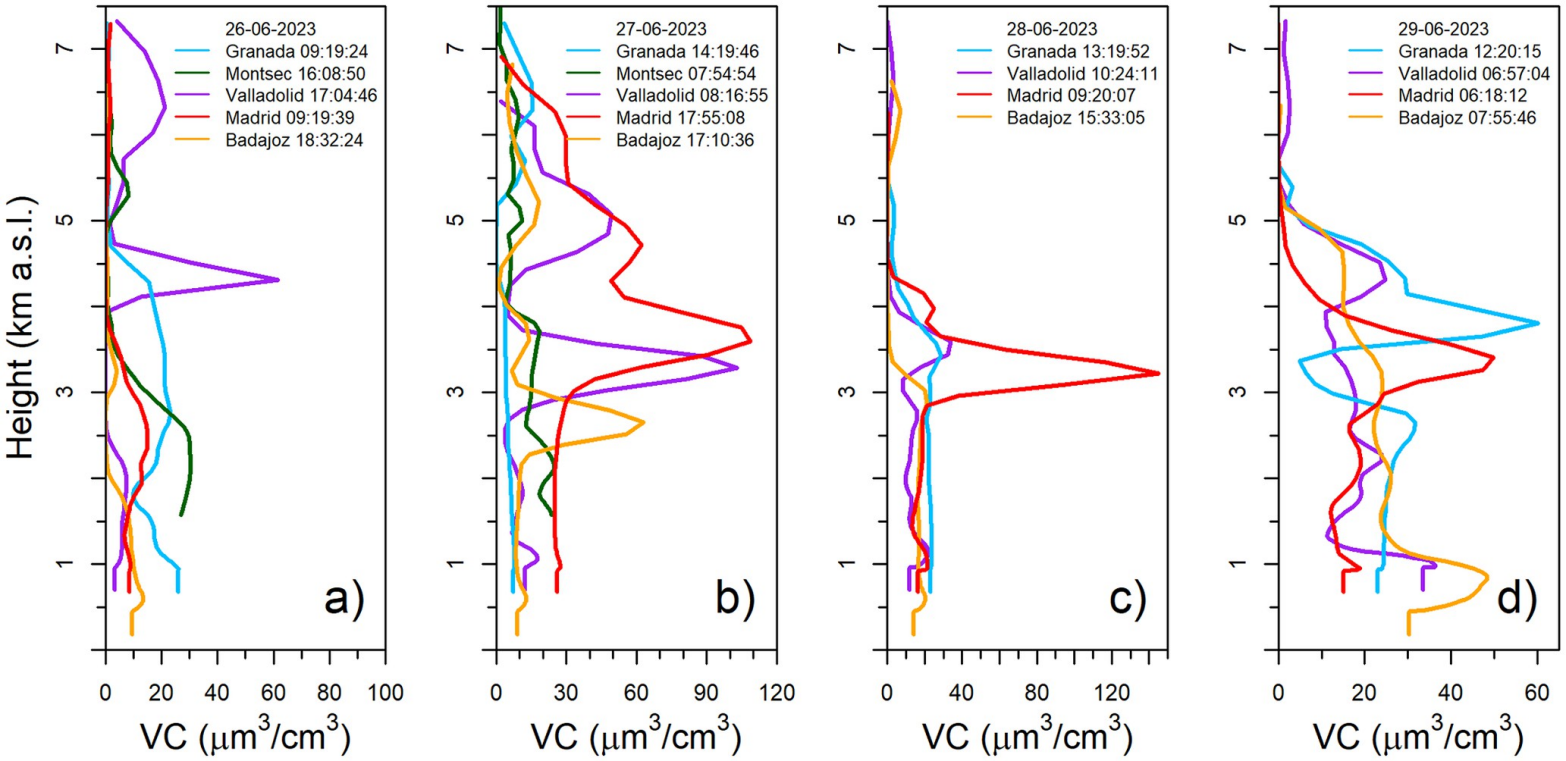
Volume concentration vertical profiles (altitudes from 0 to 7.5 km above sea level) at the studied stations. In panel a), the values are shown for June 26th, in panel b) for the 27th, panel c) for the 28th and panel d) for the 29th of June 2023, at the hours (UTC) indicated in the legend for each station.

Fig 11 shows the vertical volume concentration profiles provided by CAECENET for the days of the event. Only one retrieval is plotted in the graphs per day and station. The Valladolid VC profile on June 26th aligns well with the observed in Fig 10, showing a thin layer with a high aerosol volume concentration (up to 60 μm^3^/cm^3^) between 4 and 5 km a.s.l. and another wider layer at a higher altitude with lower concentration (about 20 μm^3^/cm^3^). In the rest of the stations on this day, there are no available inversions later, so high concentrations are not captured as the aerosol plume did not yet arrived, as seen in Fig 10. The following day, June 27th, the event is clearly observed at most of the stations. Madrid and Valladolid show similar profiles, with a concentration peak about 110 μm^3^/cm^3^, both peaks centered between 4 and 3 km a.s.l. respectively. Badajoz presents a similar peak than the mentioned for Valladolid but approximately 600 m below; this difference could be caused by the orography, since the altitude of Valladolid is 520 m above Badajoz. Another peak in a wider layer around 5 km a.s.l. can be appreciated in some locations with lower VC values: about 60, 50 and 20 μm^3^/cm^3^ in Madrid, Valladolid and Badajoz, respectively. In Granada, a layer between 6 and 7 km a.s.l. appears that day with a maximum VC value of 20 μm^3^/cm^3^, which fits with the highest layer detected in Valladolid the day before. In Montsec, there are few available observations due to clouds, but the profile shown at 07:55 UTC in Fig 11, could indicate the presence of thin layers about 2 and 5 km a.s.l.

On June 28th, a very pronounced VC peak about 140 μm^3^/cm^3^ appeared in Madrid in a thin layer around 3.2 km a.s.l. In Valladolid on this day, a thin layer is also observed around 3.5 km a.s.l. but with lower VC values, being the peak below 40 μm^3^/cm^3^. In Granada, the aerosol plume was still remaining in high altitudes but in a broader layer (between 5.5 and 7 km a.s.l.), showing values close to 30 μm^3^/cm^3^. Finally, on June 29th, a layer around 4.5 km a.s.l. can be observed in Valladolid and Badajoz, with peak VC values between 30 and 20 μm^3^/cm^3^. Granada and Madrid presented the most intense layer around 4 and 3.5 km a.s.l., respectively, with VC peak values between 60 and 50 μm^3^/cm^3^.

Regarding the vertical profiles of the extinction, scattering, absorption, and backscattering coefficients from the CAECENET inversions (shown in the S2 Fig), the same pattern as in the vertical profile of volume concentration is observed. On June 27th, Valladolid and Madrid presented the highest values of the scattering coefficient at 440 nm, 891 Mm^−1^ and 661 Mm^−1^ respectively. In Badajoz, these values were slightly lower, measuring a peak of 503 Mm^−1^. In Montsec, on 27th June, the scattering coefficient at the same wavelength reached values up to 123 Mm^−1^. In Madrid, Valladolid and Badajoz, for the altitudes mentioned in the discussion of Figs 10 and 11, the absorption coefficient at 440 nm reached a maximum of 53 Mm^−1^, 34 Mm^−1^ and 39 Mm^−1^, respectively. The maximum absorption observed in Granada was around 7 Mm^−1^ for 440 nm. The backscattering coefficient at 440 nm presented the lowest values for Granada, under 1.1 Mm^−1^sr^−1^, in the case of Madrid and Badajoz, it gets values around 7.8 and 5.5 Mm^−1^sr^−1^, and up to 12.5 Mm^−1^sr^−1^ for Valladolid. The extinction coefficient, agrees with the values mentioned for each coefficient, for 440 nm the highest values are found at Valladolid, around 940 Mm^−1^, 714 and 517 Mm^−1^ for Madrid and Badajoz respectively and around 129 and 42 Mm^−1^ for Montsec and Granada.

**Fig 12:**
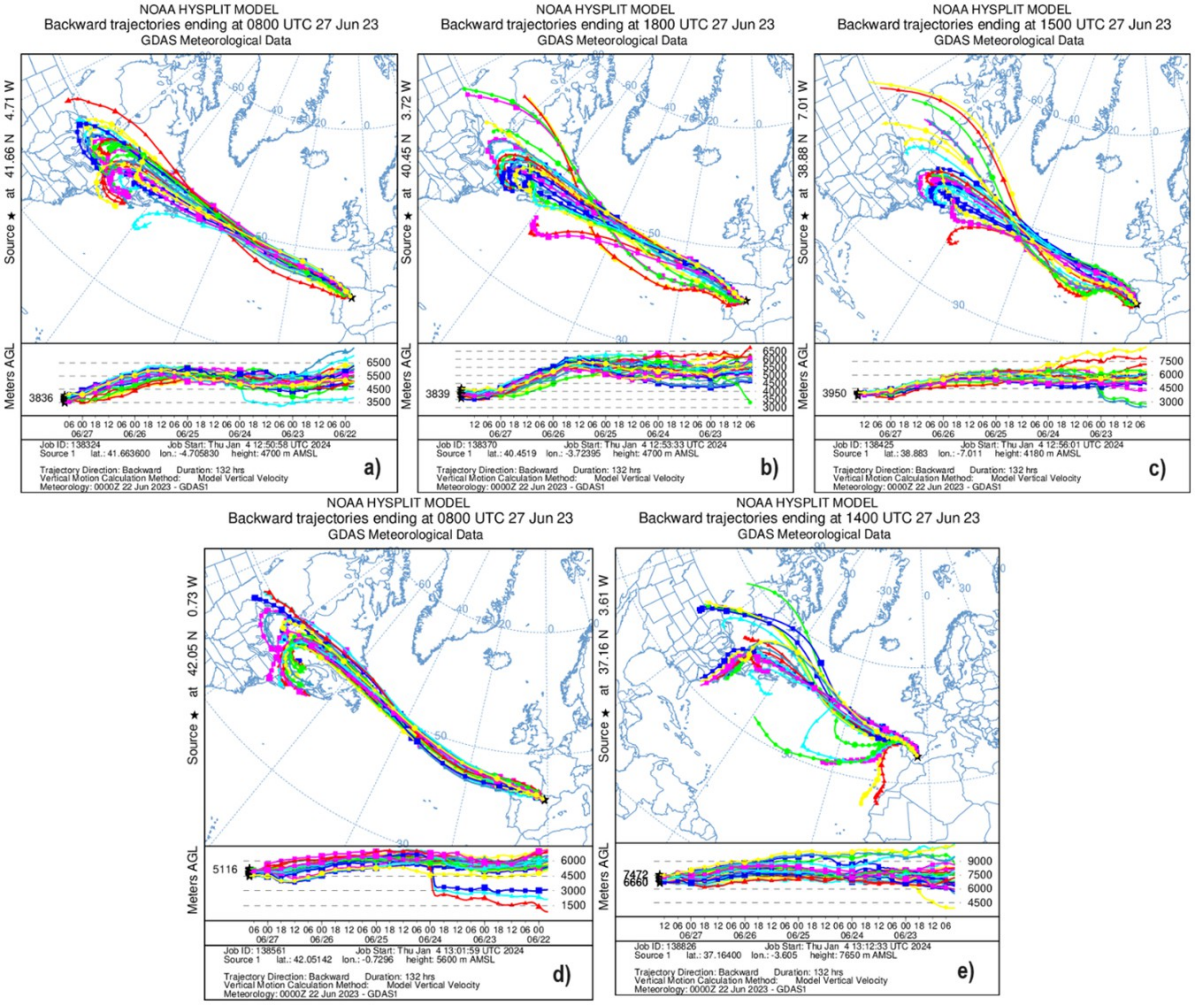
HYSPLIT back-trajectories at 4km a.s.l. from Valladolid a), Madrid b), Badajoz c), and Montsec d), and at 7 km from Granada e), on 27th June.

Once the height of aerosol layers has been quantified, the HYSPLIT model has been run using ensemble mode to calculate the back-trajectories from the analyzed stations. These back-trajectories are shown in Fig 12 and they confirm that the detected aerosol plume was transported from Canada, primarily from the region of Quebec. As shown in Fig 10, the aerosol plume was at a considerable altitude, above 6 km a.s.l., and took between 5 and 6 days to reach the Iberian Peninsula, according to back trajectories analysis. In the case of Montsec station (Fig 12d), the ensemble mode for trajectory calculation is presented to assess that air masses from the same region did reach the station. Therefore, even though there are not as many observations as in the other stations, it is possible to confirm that the event is the same. For Granada (Fig 12e), it is observed that despite the plume reached later, its origin was the same. The NCEP/DOE reanalysis shows a strong high-pressure system extending from the surface to upper levels over the Atlantic Ocean. The northward location of the Azores anticyclone together with the position of the Icelandic low favoured westerly airflow carrying aerosol from the Canadian wildfires.

## Conclusion

This work has introduced the CAECENET system, which aims to take advantage of the synergies between the data measured by collocated sun-sky photometers and ceilometers using the GRASP code in the retrieval of both column and vertical aerosol properties. The input data is obtained from CÆLIS and ICENET databases, and since both follow well-defined and well-known measurement and calibration protocols, it is possible to automatically and continuously obtain these properties in near-real-time in an accessible manner. This is very useful, for instance, in the analysis of regional aerosol events.

This study also presents the analysis of two aerosol events in the Iberian Peninsula, which have been characterized using CAECENET products. The first corresponds to a Saharan dust outbreak that occurred between October 3rd and 5th, 2022. The analyzed data have shown that on October 3rd, aerosol optical depth (AOD) at 400 nm increased up to values above 0.5 nm with Angström Exponent (AE) values below 0.2 in the Spanish stations of Madrid, Valladolid, and Granada. The vertical profiles have revealed that the layer of mineral dust aerosols was transported at around 3 km a.s.l. altitude, although in Granada, there was also aerosol load at surface level. On October 4th, the aerosols descended in altitude, being gradually removed from the atmosphere on October 5th. The optical properties provided by CAECENET fit with typical values for Saharan mineral dust particles, with SSA close to 1 except for 440 nm and very low absorption coefficients.

The other event analyzed corresponds to the transport of particles from the Canadian wildfires in June 2023. This event has been observed in the Iberian Peninsula at Badajoz, Granada, Madrid, Montsec, and Valladolid sites, where unusually high AOD values were measured between 26th and 29th June. Back-trajectories have confirmed that its origin was the wildfires in the province of Quebec in Canada. The smoke particles were transported at high altitudes to reach the Iberian Peninsula, as shown by the volume concentration profiles, with values exceeding 100 μm^3^/cm^3^. At the stations of Valladolid, Madrid, and Badajoz, aerosol plumes have been observed at different altitudes, generally between 3 and 5 km a.s.l.

Concluding, this work has demonstrated that CAECENET is a highly useful and powerful tool for monitoring, characterizing, and quantifying vertically-resolved aerosol properties. Its effectiveness has been validated through its application in previous studies, such as those investigating aerosol vertical radiative forcing [62] and monitoring volcanic plumes over the Iberian Peninsula [63].

Furthermore, in the near future, the system could be configured to integrate data from other types of lidar systems, such as Micropulse Lidars (MPLs), which offer different operational characteristics and advantages. Additionally, incorporating data from other ceilometer networks, like E-PROFILE, could expand the geographic coverage and spatial resolution of CAECENET’s observations. These advancements would enable the generation of GRASP_*pac*_ products at a greater number of locations, providing a more comprehensive understanding of aerosol properties and delivering accurate and detailed information on aerosol dynamics. All this information could be really valuable to study their impact on the environment.

## Supporting information

S1 Fig Vertical profiles (altitudes from 0 to 7.5 km above sea level) of a) extinction coefficient, b) scattering coefficient, c) absorption coefficient and d) backscattering coefficient. For October 3rd 2022, at the hours (UTC) indicated in the legend for each station.
(TIF)

S2 Fig Vertical profiles (altitudes from 0 to 7.5 km above sea level) of a) extinction coefficient, b) scattering coefficient, c) absorption coefficient and d) backscattering coefficient. For June 27th 2023, at the hours (UTC) indicated in the legend for each station.
(TIF)
