# Supplementary figures and images for: CAECENET: An automatic system processing photometer and ceilometer data from different networks to provide columnar and vertically-resolved aerosol properties
Source: PLoS One. 2024 Dec 27;19(12):e0311990. doi: 10.1371/journal.pone.0311990 (PMC11676519; doi:10.1371/journal.pone.0311990)

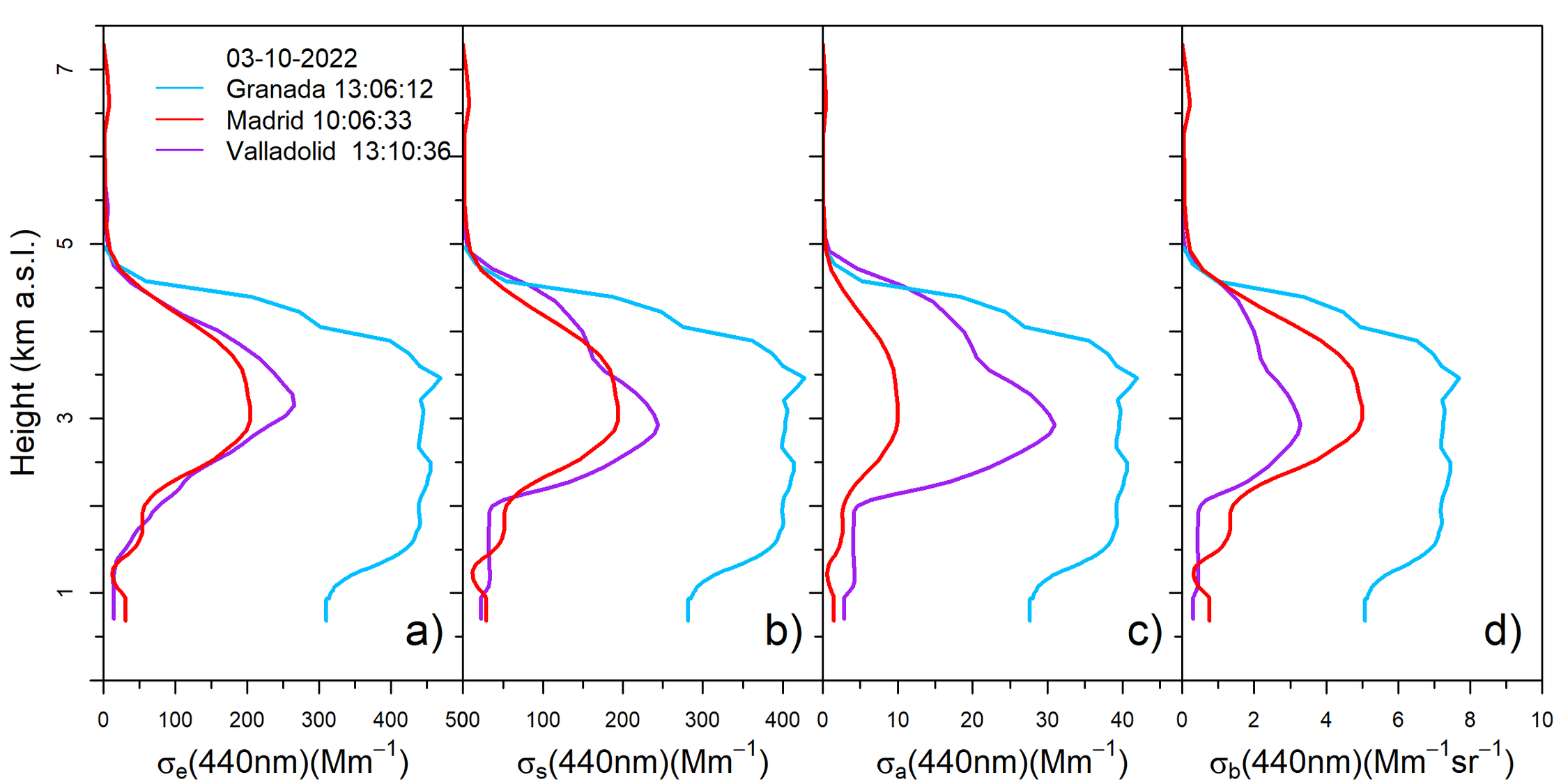

Supplement: S1 Fig — For October 3rd 2022, at the hours (UTC) indicated in the legend for each station. (TIF) [file pone.0311990.s001.tif]

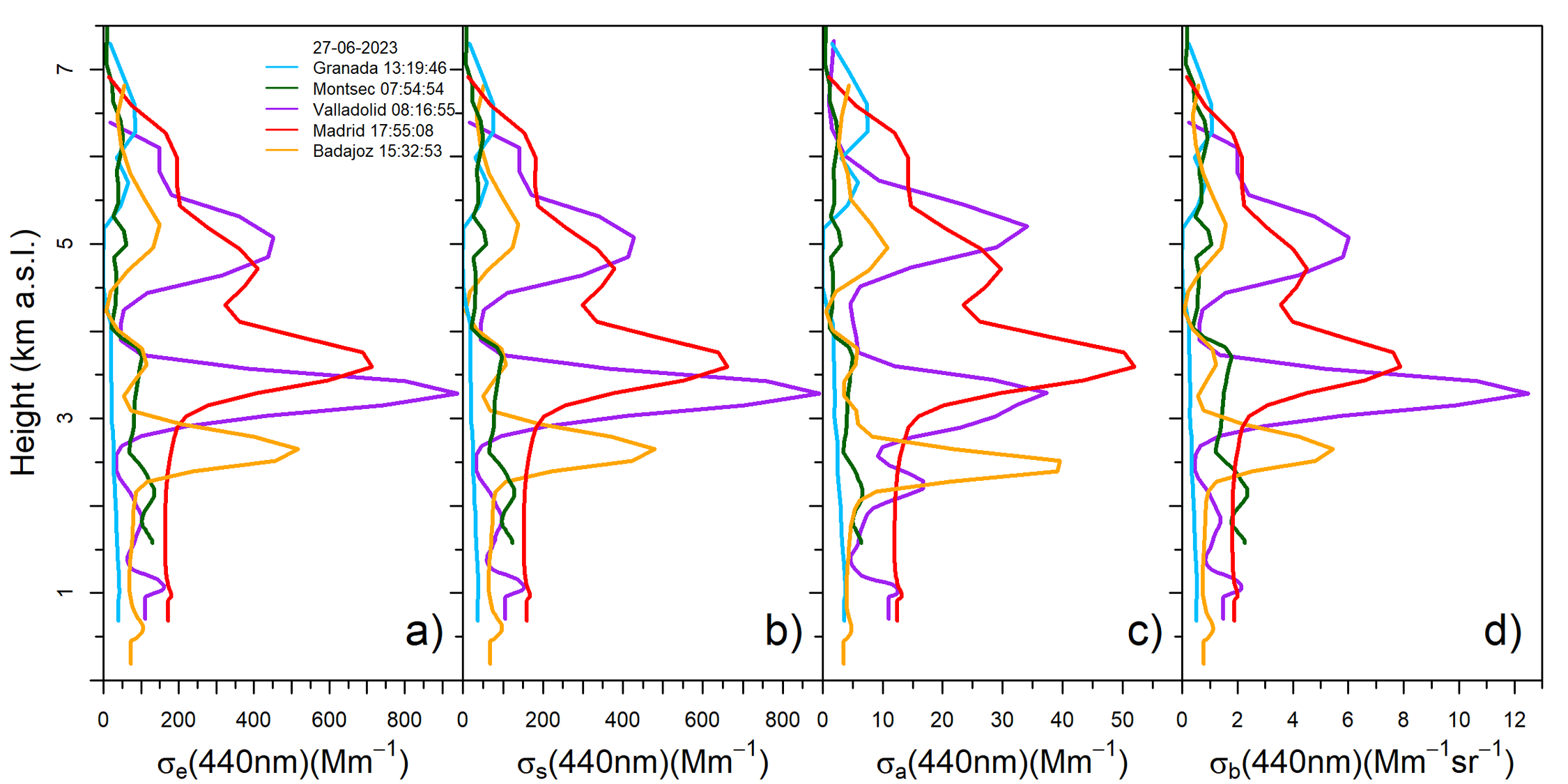

Supplement: S2 Fig — For June 27th 2023, at the hours (UTC) indicated in the legend for each station. (TIF) [file pone.0311990.s002.tif]
